# Gaps Between Guidelines and Practice in VTE Prevention for Advanced NSCLC Outpatients: A Nationwide Cross-Sectional Study in China

**DOI:** 10.3390/healthcare14070910

**Published:** 2026-04-01

**Authors:** Roujuan Wang, Qiuyi He, Jie Chen, Kejing Tang, Yubo Tang

**Affiliations:** 1 Department of Pharmacy, The First Affiliated Hospital of Sun Yat-sen University, Guangzhou 510080, China; wangrj29@mail2.sysu.edu.cn (R.W.); heqy26@mail.sysu.edu.cn (Q.H.); chenj28@mail.sysu.edu.cn (J.C.); tangkj@mail.sysu.edu.cn (K.T.); 2 School of Pharmaceutical Sciences, Sun Yat-sen University, Guangzhou 510006, China

**Keywords:** non-small-cell lung cancer, lung cancer, venous thromboembolism, thromboprophylaxis, prophylactic anticoagulation, Khorana score

## Abstract

**Highlights:**

**What are the main findings?**

- First nationwide survey reveals conservative use of VTE prophylaxis in advanced NSCLC outpatients in China, with limited adherence to guidelines.
- Direct oral anticoagulants are the most commonly preferred agents.

**What are the implications of the main findings?**

- Knowledge gaps and safety concerns are commonly perceived barriers to the implementation of thromboprophylaxis.
- Bleeding risk and monitoring challenges significantly influence clinical decision-making.

**Abstract:**

**Background**: Venous thromboembolism (VTE) is a common and serious complication in patients with advanced non-small-cell lung cancer (NSCLC). While guidelines recommend prophylactic anticoagulation for cancer outpatients at high risk, its clinical implementation remains conservative in China. **Objectives**: This study aimed to investigate the current use of prophylactic anticoagulation for advanced NSCLC outpatients at high risk of VTE in China and explore factors influencing physicians’ decision-making. **Methods**: A descriptive cross-sectional survey using a convenience sampling approach was conducted from May to June 2025 among physicians from multiple top-tier tertiary hospitals across China. The survey assessed physicians’ knowledge, practices, and concerns regarding VTE risk assessment and prophylactic anticoagulation. Descriptive statistics and multiple response analyses were performed using SPSS 25.0. **Results**: A total of 235 valid responses were collected. Although 84.7% of physicians reported receiving anticoagulation training, only 57.8% routinely used the Khorana score for risk assessment. After excluding six physicians (2.7%) who reported never assessing VTE risk, 59.4% reported initiating prophylactic anticoagulation for patients with a Khorana score ≥ 2. Direct oral anticoagulants were preferred by 75.6% of physicians. Key concerns included management of bleeding events (78.6%) and adverse reactions monitoring (61.1%). Notably, only 49.4% of physicians reported being familiar with the Khorana score. **Conclusions**: Prophylactic anticoagulation for advanced NSCLC outpatients appears to remain underutilized in China. Limited familiarity with VTE risk assessment tools and concerns regarding bleeding risk may influence physicians’ clinical decisions. Educational initiatives and prospective studies may help improve guideline adherence.

## 1. Introduction

Venous thromboembolism (VTE) remains one of the most clinically significant complications in patients with advanced non-small-cell lung cancer (NSCLC). As lung cancer is considered a highly thrombogenic malignancy, individuals with NSCLC are at particularly high risk of VTE development [1]. In advanced-stage disease, the incidence of VTE may be substantial, especially in patients receiving first-line combination chemotherapy and immunotherapy [2]. Thrombotic events are associated with treatment interruption, increased healthcare burden, and impaired survival [3].

In recent years, risk-adapted thromboprophylaxis has been proposed to reduce preventable thrombotic events in ambulatory cancer populations. Randomized clinical trials have demonstrated that prophylactic anticoagulation can significantly reduce VTE incidence in selected high-risk patients, although concerns regarding bleeding complications remain [4,5]. Several prediction models have been developed to facilitate risk stratification, among which the Khorana score is the most widely adopted and incorporated into international guidelines, including those issued by ASCO and ISTH [6,7,8]. Current guidelines do not recommend routine thromboprophylaxis for all ambulatory cancer patients but suggest its use in those at high risk of VTE, typically defined as a Khorana score ≥ 2 [7,8,9].

Despite these recommendations, the implementation of thromboprophylaxis in routine clinical practice remains inconsistent. In China, preventive anticoagulation among lung cancer patients appears conservative, even in those at elevated VTE risk [10]. Moreover, existing retrospective studies primarily describe utilization rates but provide limited insight into physicians’ awareness, decision-making processes, and perceived barriers in the outpatient management of advanced NSCLC [11].

Thromboprophylaxis decisions in ambulatory settings are largely clinician-driven and influenced by individual risk assessment and safety considerations. A physician-based survey is therefore an appropriate approach to explore current practice patterns and underlying determinants. This survey aimed to investigate the knowledge, attitudes, and practice patterns of physicians involved in the management of advanced NSCLC patients across tertiary hospitals in China.

## 2. Materials and Methods

### 2.1. Survey Respondents

A descriptive cross-sectional survey was conducted among physicians from top-tier tertiary hospitals across China who were involved in the management of advanced NSCLC. A convenience sampling approach was adopted to select potential participants. Eligible participants were required to hold valid Practicing Physician Certificates of the People’s Republic of China, be actively practicing in departments closely related to advanced NSCLC care, such as medical oncology and respiratory medicine, and provide routine clinical care to these patients. Physicians on medical leave, professional training, or otherwise absent from clinical work during the study period were excluded.

### 2.2. Survey Instrument

The questionnaire was developed based on a literature review and relevant national and international VTE prevention guidelines. The questionnaire was developed to explore the discrepancy between guideline recommendations and real-world clinical practice regarding prophylactic anticoagulation in advanced NSCLC. Items assessing anticoagulant selection were formulated with reference to current ASCO and CSCO guideline recommendations. Knowledge-related questions were constructed based on the Khorana risk assessment model to evaluate physicians’ understanding of VTE risk stratification.

The questionnaire comprised 19 items covering the following domains:

1. Physician demographics and professional characteristics;
2. Training experience in anticoagulation;
3. VTE risk assessment practices, including the use of the Khorana score;
4. Prophylactic anticoagulation decision-making, anticoagulant preferences and selection criteria;
5. Physicians’ concerns about anticoagulation;
6. Physicians’ knowledge of the Khorana score.

Different response formats were applied according to the content of each domain. Most demographic and professional characteristics were assessed using single-choice questions, while training channels, anticoagulation concerns, and factors influencing drug selection allowed multiple responses. Attitudes and clinical decision-making preferences (e.g., performing VTE risk assessment) were evaluated using single-choice Likert-type questions. Two single-choice knowledge questions were included to objectively assess physicians’ understanding of the Khorana risk assessment model: one addressing the lung cancer-specific score and the other assessing the recommended threshold for initiating prophylactic anticoagulation. These knowledge items were scored in a binary manner (correct = 1, incorrect = 0), and accuracy rates were calculated as the proportion of correct responses.

Prior to formal distribution, the questionnaire was pilot-tested among 20 physicians to evaluate clarity, feasibility, and internal consistency. The pilot results demonstrated acceptable reliability (Cronbach’s α = 0.727) and sampling adequacy (KMO measure = 0.711; Bartlett’s test of sphericity, *p* < 0.001). As no major issues were identified during the pilot testing, the questionnaire content remained unchanged and was subsequently finalized for nationwide dissemination.

### 2.3. Survey Procedure

After obtaining ethical approval from the Ethics Committee of The First Affiliated Hospital of Sun Yat-sen University (No. [2025]291), the questionnaire was disseminated using a professional online platform (Wenjuanxing, www.wjx.cn). A QR code linking to the survey was distributed nationwide. It should be noted that as the questionnaire link was disseminated broadly without a predefined sampling frame, the total number of physicians who received the invitation could not be determined; therefore, a response rate could not be calculated.

To ensure data quality, each device was restricted to a single submission. The collected information, with the exception of the hospital’s regional location for the purpose of ensuring nationwide coverage, contained no personal identifiers. All responses were strictly anonymous and analyzed in an aggregated manner. The survey was conducted between May and June 2025.

### 2.4. Statistical Analysis

Data were analyzed using SPSS version 25.0 (IBM Corp., Armonk, NY, USA). The reliability and validity of the questionnaire were assessed using Cronbach’s α, the Kaiser–Meyer–Olkin (KMO) measure, and Bartlett’s test of sphericity. Descriptive statistics were applied to summarize categorical variables (e.g., physician title, department, education) as frequencies and percentages. Multiple response analysis was employed for questions with multiple-choice options (e.g., training channels, anticoagulation concerns).

Given that the survey primarily aimed to describe physicians’ knowledge, attitudes, and self-reported practices rather than to evaluate causal determinants of clinical decision-making, the analysis focused mainly on descriptive statistics.

### 2.5. Reporting Guideline

This study was conducted and reported in accordance with the Strengthening the Reporting of Observational Studies in Epidemiology (STROBE) guidelines for cross-sectional studies.

## 3. Results

### 3.1. Survey Response and Questionnaire Reliability

A total of 235 valid responses were collected. The survey demonstrated a broad geographical coverage, encompassing physicians from 17 provinces across China. The participant cohort exhibited considerable diversity, representing a wide spectrum of professional titles, clinical experience, and departmental affiliations. The final questionnaire demonstrated good internal consistency (Cronbach’s α = 0.749) and structural validity (KMO measure = 0.795; Bartlett’s test of sphericity, *p* < 0.001), confirming its suitability for the subsequent analyses.

### 3.2. Characteristics of Participating Physicians

Table 1 summarizes the demographic and professional characteristics of the participating physicians. Among the respondents, attending physicians accounted for the largest proportion (41.7%), followed by associate chief physicians (30.6%), resident physicians (20.4%), and chief physicians (7.2%). Respondents were primarily from oncology (55.7%) and respiratory medicine departments (33.6%), with 10.6% from other departments such as thoracic surgery, radiation oncology and lung cancer centers.

Regarding clinical experience, 27.2% had practiced for 5–10 years, followed by <5 years (23.4%), 10–15 years (23.4%), 15–20 years (18.7%), and >20 years (7.2%). The majority (58.3%) held a master’s degree, followed by a bachelor’s degree (23.8%) and a doctoral degree (17.4%).

### 3.3. Anticoagulation Training and Knowledge

**Figure 1:**
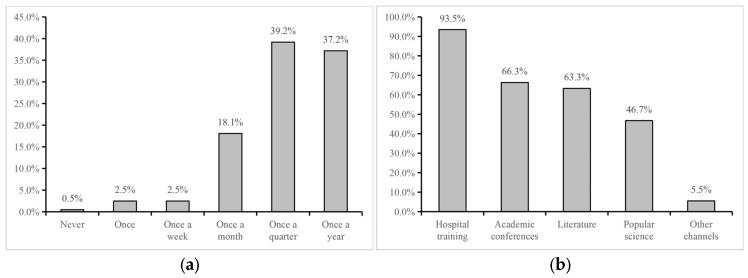
Results on the anticoagulation training of physicians. (**a**) Training frequencies (single-choice question); (**b**) Training channels (multiple-response question). Percentages in panel (**b**) represent the proportion of respondents selecting each option and may not sum to 100%.

As shown in Figure 1, 84.7% of physicians reported they had received anticoagulation training, most commonly on a quarterly (39.2%) or annual (37.2%) basis. Hospital-based training (93.5%) was the predominant format, followed by academic conferences (66.3%) and literature review (63.3%).

**Figure 2:**
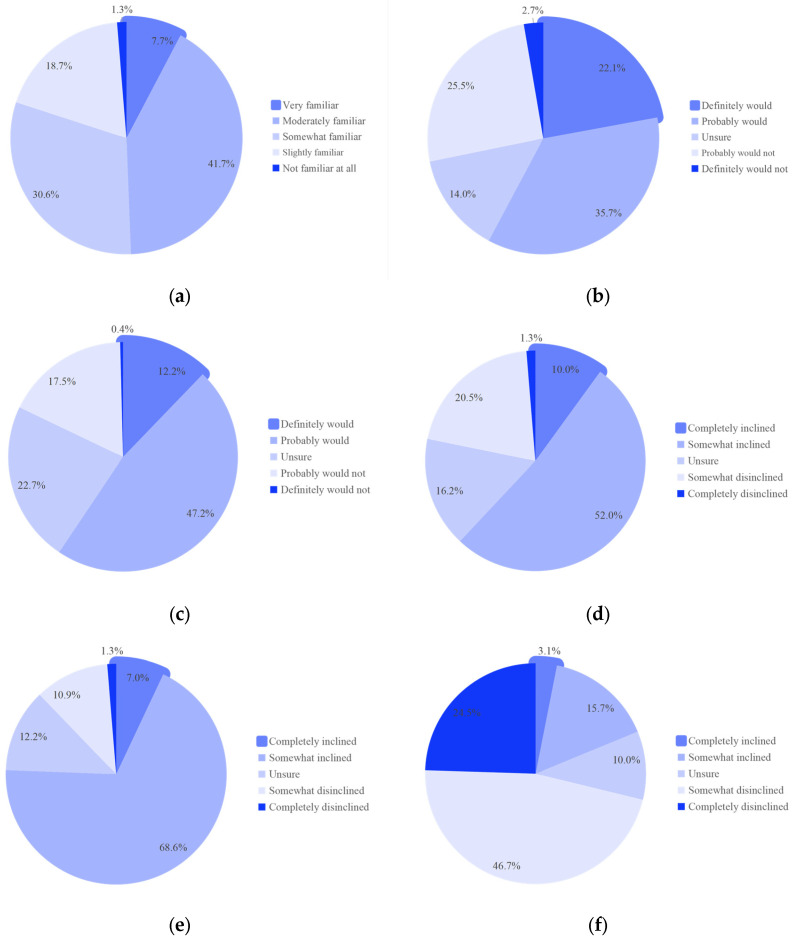
Results on the theoretical knowledge evaluation and practical application for prophylactic anticoagulation of physicians: (**a**) Self-evaluation of understanding of the Khorana score. (**b**) Answer on whether Khorana scoring will be performed on advanced NSCLC ambulatory patients undergoing chemotherapy. (**c**) Answer on whether physicians will carry out prophylactic anticoagulation for advanced NSCLC outpatients undergoing chemotherapy with a Khorana score of ≥2. (**d**–**f**) Answers on whether physicians are inclined to choose which drug for prophylactic anticoagulation: (**d**) LMWH, (**e**) DOACs, (**f**) warfarin. NSCLC, non-small-cell lung cancer; LMWH, low molecular weight heparin; DOACs, direct oral anticoagulants. Percentages are rounded to one decimal place, and minor adjustments were made to ensure the total sums to 100%.

However, gaps between training and knowledge remained significant. As shown in Figure 2a, although 49.4% of respondents claimed being familiar with the Khorana score, merely 7.7% considered themselves very familiar. Furthermore, specific questions were designed to objectively evaluate physicians’ knowledge. The assessments revealed accuracy rates of 55.3% for the lung cancer-specific score and 65.1% for the prophylactic anticoagulation threshold.

### 3.4. Clinical Practice Patterns

Figure 2b,c illustrate real-world prophylactic anticoagulation practices. Only 57.8% of physicians routinely used the Khorana score to assess VTE risk in advanced NSCLC outpatients undergoing chemotherapy. Excluding six physicians (2.7%) who have never evaluated VTE risk, 59.4% reported initiating prophylactic anticoagulation in patients with a Khorana score of ≥2.

**Figure 3:**
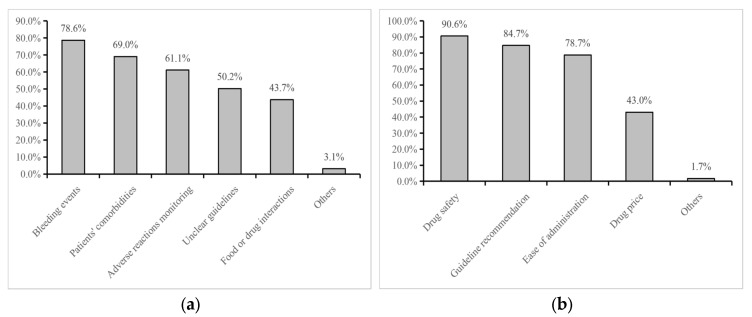
Factors influencing practice patterns of physicians. (**a**) Anticoagulation concerns (multiple-response question); (**b**) Anticoagulants selection criteria (multiple-response question). Percentages in panel (**a**,**b**) represent the proportion of respondents selecting each option and may not sum to 100%.

In terms of anticoagulant selection, most physicians preferred direct oral anticoagulants (DOACs, 75.6%, Figure 2e), followed by low molecular weight heparin (LMWH, 62.0%, Figure 2d) and warfarin (18.8%, Figure 2f). Safety (90.6%) and guideline recommendations (84.7%) were the primary determinants influencing drug selection (Figure 3b).

### 3.5. Physicians’ Concerns Regarding Anticoagulation

Figure 3a summarizes major barriers to prophylactic anticoagulation. The leading concerns were the management of bleeding events (78.6%), the complexity of patient comorbidities (69.0%), and difficulties in monitoring adverse reactions (61.1%). Additional considerations included insufficiently clear guidelines (50.2%), potential drug–drug interactions (43.7%), and procedural interventions such as surgery or biopsy (3.1%).

## 4. Discussion

This nationwide survey provides the first comprehensive assessment of prophylactic anticoagulation practices for advanced NSCLC outpatients undergoing chemotherapy at high risk of VTE in China.

### 4.1. Conservative Use of Prophylactic Anticoagulation

Although 84.7% of physicians had received anticoagulation training, the reported use of the Khorana score for VTE risk stratification and the initiation of prophylactic anticoagulation when the Khorana score was ≥2 appeared relatively limited.

Similar implementation patterns have been described in previous studies. A single-center study in China reported that only 8.3% of lung cancer patients at high risk of VTE received prophylactic anticoagulation [10]. A national survey in 2019 found that 9.5% of healthcare providers routinely administered prophylactic anticoagulation for non-surgical critically ill cancer patients [11].

International data from the Edwards Comprehensive Cancer Center further demonstrated a comparable pattern: although 34% of patients were identified as having a Khorana score ≥2 and were therefore eligible for prophylactic anticoagulation, only 7% actually received anticoagulation therapy. Notably, the reasons for withholding prophylaxis were not documented in many cases [12].

Collectively, these findings suggest that the translation of guideline-based risk stratification into routine oncology practice may remain variable across different healthcare settings.

### 4.2. Physician Knowledge and Risk-Benefit Concerns

Only half of the respondents reported familiarity with the Khorana score, and the accuracy rates for key scoring questions were below 70%. Consistent with our findings, a cross-sectional descriptive study reported that additional cost, fear of bleeding and lack of knowledge on thromboprophylaxis use (50%, 38.5% and 30.8% respectively) were commonly perceived barriers to its use [13]. These findings suggest a notable gap in knowledge translation from guidelines to clinical practice.

In the context of high VTE incidence and poor prognosis in advanced NSCLC, despite guideline recommendations supporting risk-adapted prevention strategies, bleeding risk remained the most frequently reported concern among physicians (78.6%). In this survey, knowledge gaps and safety concerns were identified as perceived barriers to thromboprophylaxis. However, these factors were based on self-reported responses and were not evaluated as independent predictors of clinical behaviour. Therefore, educational initiatives and implementation strategies may help address these concerns and facilitate more informed decision-making.

### 4.3. Anticoagulant Selection and Influence Factors

In our study, DOACs were the most preferred anticoagulants, surpassing LMWH and warfarin, which offer the advantages of oral administration and do not require routine monitoring. Moreover, the preference is likely driven by two key factors. On one hand, the clinical benefits of DOACs have been validated in two large randomized controlled trials—AVERT [4] and CASSINI [5]—which demonstrated that prophylactic rivaroxaban or apixaban significantly reduced the incidence of VTE in high-risk ambulatory cancer patients compared with placebo; on the other hand, major international guidelines, including those from ASCO and ISTH, have endorsed DOACs as recommended agents for thromboprophylaxis in cancer patients with Khorana scores of ≥2.

Importantly, physicians in our survey prioritized safety as the top factor influencing anticoagulant selection, highlighting the need for real-world studies to evaluate the safety and efficacy of DOACs for thromboprophylaxis in Chinese oncology populations.

### 4.4. Future Measures

Based on the reported knowledge gaps and safety concerns identified in this survey, training programs focusing on both VTE risk assessment and bleeding events management may improve physicians’ familiarity with guideline-recommended strategies. Such initiatives may also increase confidence in clinical decision-making. Strengthening physicians’ familiarity with the Khorana score and enhancing confidence in managing bleeding-related complications may reduce hesitation in initiating prophylactic anticoagulation.

Beyond individual training, institutional culture may play a critical role in shaping clinical decision-making. Establishing multidisciplinary collaboration frameworks, standardized thromboprophylaxis protocols, and structured bleeding management pathways may provide physicians with greater clinical support. Active engagement from institutional leadership, particularly through integrating VTE prevention into routine clinical governance and quality improvement initiatives, may further promote adherence to evidence-based practice.

Finally, future studies are warranted to generate real-world data on the safety and effectiveness of prophylactic anticoagulation in Chinese oncology populations. In parallel, refinement of current risk prediction models through the incorporation of patient-specific factors (e.g., comorbidities and laboratory parameters) may facilitate more individualized decision-making, thereby improving patient outcomes while minimizing adverse events.

## 5. Conclusions

This national survey highlights the apparent underutilization of prophylactic anticoagulation among advanced NSCLC outpatients at high risk of VTE in China, which appears to be associated with physicians’ knowledge gaps and safety concerns. Addressing these barriers through targeted education, robust real-world evidence, and improved risk assessment models may help align clinical practice with international standards and support more informed clinical decision-making.

### Strengths and Limitations

As the first national survey on prophylactic anticoagulation in advanced NSCLC outpatients with a Khorana score ≥ 2, this study provides valuable insights into real-world practice patterns and physician awareness. The large sample size and broad geographic coverage enhance the representativeness of the findings, and the questionnaire demonstrated good internal consistency and structural validity.

Nonetheless, several limitations should be acknowledged.

First, the cross-sectional and self-reported design is susceptible to social desirability and recall bias. The reported practices reflect physicians’ perceptions rather than objectively verified prescribing behaviours.

Second, because the questionnaire was disseminated without a predefined sampling frame, the total number of invited physicians was unknown and a response rate could not be calculated. As a result, the representativeness of the sample cannot be fully evaluated. In addition, participants were primarily from top-tier tertiary hospitals, which may limit generalizability to physicians practicing in secondary or primary healthcare settings. Potential self-selection bias may also exist, as physicians with a greater interest in VTE management may have been more likely to participate.

Third, this study did not include patient-level clinical characteristics, contraindications to anticoagulation, or outcome data. Therefore, the survey could not capture individualized risk–benefit considerations in real-world practice, nor could the correlation between reported practices and actual patient outcomes be evaluated.

Furthermore, due to the relatively limited sample size, exploratory subgroup analyses stratified by physician characteristics (e.g., years of clinical experience or departmental affiliation) were not performed. Future large-scale studies incorporating patient-level data are warranted to further validate and extend these findings.

## Figures and Tables

**Table 1:** Survey variable table.

Category	Variables	Responses	Number of Responses (%)
Demographics	Title	Resident physician	48 (20.4)
		Attending physician	98 (41.7)
		Associate chief physician	72 (30.6)
		Chief physician	17 (7.2)
	Department	Respiratory department	79 (33.6)
		Oncology department	131 (55.7)
		Other departments	25 (10.6)
	Time in practice	<5 years	55 (23.4)
		5–10 years	64 (27.2)
		10–15 years	55 (23.4)
		15–20 years	44 (18.7)
		>20 years	17 (7.2)
	Highest educational qualification	Below bachelor	1 (0.4)
		Bachelor	56 (23.8)
		Master	137 (58.3)
		Doctor	41 (17.4)
Anticoagulation training	Anticoagulation training experience	Yes	199 (84.7)
		No	36 (15.3)
	Anticoagulation training frequency	Never	1 (0.5)
		Once	5 (2.5)
		Once a week	5 (2.5)
		Once a month	36 (18.1)
		Once a quarter	78 (39.2)
		Once a year	74 (37.2)
	Training channels (multiple-response question)	Hospital training	186 (93.5)
		Academic conferences	132 (66.3)
		Literature	126 (63.3)
		Popular science	93 (46.7)
		Other channels	11 (5.5)
Knowledge measures	Familiarity with Khorana score	Very familiar	18 (7.7)
		Moderately familiar	98 (41.7)
		Somewhat familiar	72 (30.6)
		Slightly familiar	44 (18.7)
		Not familiar at all	3 (1.3)
	Lung cancer-specific score (“1 point” is correct)	0 points	2 (0.9)
		1 point	130 (55.3)
		2 points	79 (33.6)
		3 points	23 (9.8)
		4 points	1 (0.4)
	Prophylactic anticoagulation threshold (“2 points” is correct)	0 points	3 (1.3)
		1 point	13 (5.5)
		2 points	153 (65.1)
		3 points	57 (24.3)
		4 points	9 (3.8)
Clinical Practice Patterns	Use of Khorana score	Definitely would	52 (22.1)
		Probably would	84 (35.7)
		Unsure	33 (14.0)
		Probably would not	60 (25.5)
		Definitely would not	6 (2.7)
	Initiation of prophylactic anticoagulation when Khorana score ≥ 2	Definitely would	28 (12.2)
		Probably would	108 (47.2)
		Unsure	52 (22.7)
		Probably would not	40 (17.5)
		Definitely would not	1 (0.4)
	Anticoagulant selection—LMWH	Completely inclined	23 (10.0)
		Somewhat inclined	119 (52.0)
		Unsure	37 (16.2)
		Somewhat disinclined	47 (20.5)
		Completely disinclined	3 (1.3)
	Anticoagulant selection—DOACs	Completely inclined	16 (7.0)
		Somewhat inclined	157 (68.6)
		Unsure	28 (12.2)
		Somewhat disinclined	25 (10.9)
		Completely disinclined	3 (1.3)
	Anticoagulant selection—warfarin	Completely inclined	7 (3.1)
		Somewhat inclined	36 (15.7)
		Unsure	23 (10.0)
		Somewhat disinclined	107 (46.7)
		Completely disinclined	56 (24.5)
Factors influencing clinical practice	Anticoagulation concerns (multiple-response question)	Bleeding events	180 (78.6)
		Patients’ comorbidities	158 (69.0)
		Adverse reactions monitoring	140 (61.1)
		Unclear guidelines	115 (50.2)
		Food or drug interactions	100 (43.7)
		Others	7 (3.1)
	Anticoagulant selection criteria (multiple-response question)	Drug safety	213 (90.6)
		Guideline recommendation	199 (84.7)
		Ease of administration	185 (78.7)
		Drug price	101 (43.0)
		Others	4 (1.7)

## Data Availability

The raw data supporting the conclusions of this article will be made available by the authors on request.

## Abbreviations

The following abbreviations are used in this manuscript:

VTE	Venous thromboembolism
NSCLC	Non-small-cell lung cancer
ISTH	International Society for Thrombosis and Hemostasis
ASCO	American Society of Clinical Oncology
KMO	Kaiser–Meyer–Olkin
STROBE	Strengthening the Reporting of Observational Studies in Epidemiology
LMWH	Low molecular weight heparin
DOACs	Direct oral anticoagulants
AVERT	Apixaban for the Prevention of Venous Thromboembolism in High-Risk Ambulatory Cancer Patients trial
CASSINI	Rivaroxaban Thromboprophylaxis in High-Risk Ambulatory Cancer Patients Receiving Systemic Therapy: Results of a Randomized Clinical Trial

## References

[1] Zer A., Moskovitz M., Hwang D.M., Hershko-Klement A., Fridel L., Korpanty G.J., Dudnik E., Peled N., Shochat T., Leighl N.B. (2017). ALK -Rearranged Non–Small-Cell Lung Cancer Is Associated with a High Rate of Venous Thromboembolism. Clin. Lung Cancer.

[2] Aguiar De Azevedo L., Orione C., Tromeur C., Couturaud F., Descourt R., Geier M. (2024). Incidence of Venous Thromboembolism and Association with PD-L1 Expression in Advanced Non-Small Cell Lung Cancer Patients Treated with First-Line Chemo-Immunotherapy. Front. Oncol..

[3] Zhang M., Wu S., Hu C. (2020). Do Lung Cancer Patients Require Routine Anticoagulation Treatment? A Meta-Analysis. J. Int. Med. Res..

[4] Carrier M., Abou-Nassar K., Mallick R., Tagalakis V., Shivakumar S., Schattner A., Kuruvilla P., Hill D., Spadafora S., Marquis K. (2019). Apixaban to Prevent Venous Thromboembolism in Patients with Cancer. N. Engl. J. Med..

[5] Khorana A.A., Soff G.A., Kakkar A.K., Vadhan-Raj S., Riess H., Wun T., Streiff M.B., Garcia D.A., Liebman H.A., Belani C.P. (2019). Rivaroxaban for Thromboprophylaxis in High-Risk Ambulatory Patients with Cancer. N. Engl. J. Med..

[6] Khorana A.A., Kuderer N.M., Culakova E., Lyman G.H., Francis C.W. (2008). Development and Validation of a Predictive Model for Chemotherapy-Associated Thrombosis. Blood.

[7] Key N.S., Khorana A.A., Kuderer N.M., Bohlke K., Lee A.Y.Y., Arcelus J.I., Wong S.L., Balaban E.P., Flowers C.R., Gates L.E. (2023). Venous Thromboembolism Prophylaxis and Treatment in Patients with Cancer: ASCO Guideline Update. J. Clin. Oncol..

[8] Wang T., Zwicker J.I., Ay C., Pabinger I., Falanga A., Antic D., Noble S., Khorana A.A., Carrier M., Meyer G. (2019). The Use of Direct Oral Anticoagulants for Primary Thromboprophylaxis in Ambulatory Cancer Patients: Guidance from the SSC of the ISTH. J. Thromb. Haemost..

[9] Farge D., Frere C., Connors J.M., Khorana A.A., Kakkar A., Ay C., Muñoz A., Brenner B., Prata P.H., Brilhante D. (2022). 2022 International Clinical Practice Guidelines for the Treatment and Prophylaxis of Venous Thromboembolism in Patients with Cancer, Including Patients with COVID-19. Lancet Oncol..

[10] Zou H., Tian D., Liu C., Xiao J., Wang P., Zhang Y. (2018). Venous Thromboembolism and Prophylactic Anticoagulation Therapy in Patients with Lung Cancer: A Survey and Analysis. J. Clincal Intern. Med..

[11] Xu J., Wang H. (2019). The Current Knowledge and Execution Status of Venous Thromboembolism Prevention and Management among Critical Care Practitioners of Cancer Hospitals in China: A Muticenter Survery. Chin. J. Integr. Tradit. West. Med. Intensive Crit. Care.

[12] Khan N.A.J., Yasin H., Nwanwene K., Abdallah M., Gress T., Tirona M. (2021). Assessment of Venous Thromboembolism Risk Based on the Khorana Predictive Model and Compliance with Prophylactic Anticoagulation at a Regional Cancer Center- a Quality Improvement Study. Blood.

[13] Ekwere T., Ino-Ekanem B., Ekanem A. (2015). Venous Thromboembolism: Awareness and Practice of Thromboprophylaxis among Physicians in a Tertiary-Care Hospital. Int. J. Med. Biomed. Res..

